# Artificial Intelligence in Spiritual Care: Modified Delphi Study

**DOI:** 10.2196/97094

**Published:** 2026-07-27

**Authors:** Fabian Winiger, C Estelle Smith, Shadi Nourriz, Csaba Szilagyi, Annette Haußmann

**Affiliations:** 1 URPP Digital Religion(s) University of Zurich Zurich Switzerland; 2 Department of Computer Science Colorado School of Mines Golden, CO United States; 3 Transforming Chaplaincy Rush University Chicago, IL United States; 4 Faculty of Theology Heidelberg University Heidelberg, Baden-Wurttemberg Germany

**Keywords:** AI, artificial intelligence, chaplaincy, consensus, Delphi, health care, professional competence, spiritual care

## Abstract

**Background:**

Spiritual care providers are increasingly challenged to address the introduction of AI within the ethical, theological, and organizational bounds of their employers and religious or worldview communities. However, empirical data on professional perspectives regarding AI implementation in spiritual care remain scarce.

**Objective:**

This study aimed to conduct the first empirical investigation of AI use cases, risks and benefits, theological and ethical considerations, and relevant professional competencies from a multistakeholder expert perspective.

**Methods:**

An international, multistakeholder modified Delphi study was conducted in 2 rounds. A purposive sample of 149 subject experts was recruited. Panelists rated 213 items spanning task assistance and substitution, risks and benefits, theological and ethical considerations, limits, and competencies. Consensus was defined using combined measures of variance and directionality. Exploratory subgroup analyses assessed whether ratings differed across professional and demographic groups.

**Results:**

Round 1 was completed by 102 of 149 invited panelists (response rate 68.5%); round 2 was completed by 83 panelists (response rate 81.4%). In round 2, strong agreement emerged that AI can currently assist with or enhance administrative and routine tasks (77/81, 95.1%), informational tasks (74/79, 93.7%), documentation (67/80, 83.8%), and spiritual care research (65/77, 84.4%). Agreement was lower for relational, patient-facing tasks such as creating supportive spaces (30/69, 43.5%), direct patient engagement (32/76, 42.1%), and conducting ritual tasks (32/76, 42.1%). Panelists favored AI assistance over substitution and rated the future potential of AI above its current capabilities. The highest-ranked benefit that reached consensus was improved screening and triage; the highest-ranked risk was loss of human connection. Overall, 45.8% (38/83) of panelists judged the benefits of AI in spiritual care to outweigh the risks. The panel converged strongly on professional limits but was more divided on the underlying theological and ethical objections. The most strongly endorsed competencies were judging AI’s applicability and the boundaries of its use, safeguarding patient privacy and data, and assessing and mitigating risks. Subgroup analyses produced few robust differences.

**Conclusions:**

This study provides the first task-level map of expert opinion on AI in spiritual care, suggesting that current expert views are task-specific, future-oriented, and conditional. AI is seen as capable of assisting with administrative, informational, documentation, and research tasks. Direct relational care is widely regarded as a primarily human responsibility, and several tasks that experts judged AI capable of assisting with or substituting remain ethically complex. Experts converged on practical safeguards, including human oversight, evidence-based implementation, privacy protection, and limits on AI decision-making, and on required competencies, even as the theological and ethical rationales behind them remained contested. This suggests that professional guidance may be achievable at an early stage of AI adoption and can inform future research, guideline development, and curriculum design.

## Introduction

### Overview

Spiritual care is a professional clinical discipline that integrates patients’ religious, spiritual, and existential needs into interdisciplinary, whole-person patient care. Spiritual care providers are often called chaplains and have graduate education and extensive training in Clinical Pastoral Education [1]. They may conduct comprehensive spiritual assessments, develop care plans, facilitate ethical decision-making to align treatment with patient values, and provide process- and resource-oriented care to patients and their relatives. Attention to patients’ beliefs, values, and practices reflects a broader shift toward treating religion and spirituality as relevant dimensions of health care and global health [2,3]. In this study, AI is understood to include generative systems such as large language models and classification- and decision-making systems. In health care, these are increasingly used in screening, assessment, and documentation, as well as in chatbot applications for patient interactions.

Spiritual care providers are increasingly challenged to address the introduction of AI in health care settings within the ethical, theological, and professional bounds of their employers and religious or worldview communities. However, there is little empirical research regarding the appropriate use of AI in professional spiritual care settings. This creates uncertainty about how emerging AI applications should be evaluated in relation to patient needs, professional standards, and the theological formation and ethical discernment of care providers. This study responds to this research gap with an international, multistakeholder Delphi study among board-certified spiritual care providers, department managers, academic leaders, and experts in the ethics and theology of AI (round 1: N=102; round 2: N=83).

The Delphi process was chosen due to its suitability for exploring expert opinion on emerging phenomena and informing best practice in the medical field. Building on existing research on AI and spiritual care, this study aims to map current opinions among the professional community regarding the use of AI in professional spiritual care across 5 domains: assisting with, enhancing, or substituting specific tasks; associated risks and benefits; theological and ethical considerations; appropriate limits; and required competencies for safe and effective AI use by spiritual care providers.

### Background

AI is becoming increasingly relevant for spiritual care providers. This development is related to 2 factors. First, the use of AI for religious and spiritual self-care purposes is increasing. Contrary to claims by leading AI companies such as OpenAI and Anthropic that the percentage of messages for social and emotional purposes is in the low single digits [4,5], there are mounting reports of users engaging in relationships with chatbots, and online communities dedicated to AI companionship have reached tens of thousands of members [6,7]. Data published by advocacy and policy advisory groups suggest that one-third of the British population uses chatbots for mental health support [8,9]. While some evidence suggests that chatbots may have positive effects on mental health [10], concerns have emerged that prolonged chatbot use may exacerbate mental disorders and symptoms such as affective disorders or suicidality and trigger severe psychiatric episodes [11-13]. Some mental health professionals have proposed the terms “AI psychosis” and “chatbot psychosis” to describe this phenomenon [14-17].

Chatbot-induced delusional episodes may be religiously connoted and revolve around narratives in which the chatbot claims to mediate between the user and a supposed mystical, sacred, or transcendental dimension or suggests that the user has attained, or by means of the chatbot can attain, access to a hidden spiritual reality [18-20]—or that the chatbot itself represents a spiritually significant entity [21,22]. Religious chatbots developed by religious communities and private-sector entrepreneurs are often presented as tools that may facilitate spiritual or religious experiences [23,24]. Specialized “griefbots,” which use the data of deceased persons to generate outputs, are claimed to help with managing grief [25]. In health care settings, both of these tasks traditionally lie within the remit and expertise of qualified spiritual care providers.

A second factor in the growing relevance of AI to spiritual care providers is the rapid development and adoption of AI-enabled medical technologies by health care providers, in particular in screening and assessment, clinical decision-making, charting, and patient communication. For example, ambient AI scribes record interactions with patients for charting purposes [26]. Generative AI applications are also developed for patient communication and mental and psychosocial patient care [27,28].

Spiritual care providers are challenged to respond to these developments. In some US-based health systems, chaplains have begun to formulate ethical and professional positions regarding the use of AI in their work and participate in developing AI-enabled technologies sensitized to the spiritual needs of patients and professional requirements of spiritual care providers. The Association of Professional Chaplains (APC) has published ethical guidelines on AI use in its board-certification process [29] and recently launched ETHOS (Ethical Technology for Human Oriented Spiritual Care), a task force to develop ethical guidelines for best practice in the use of AI. A literature on spiritual care and AI has emerged, centered on the question of whether AI-enabled chatbots are similar to human care providers and, relatedly, the extent to which they may be used to provide direct care [24,30-32]. While more recent studies have investigated the relevance of AI in relation to specific use cases such as spiritual emergency care [33] and chaplaincy education [34], these studies are based on anecdotal reports or small experimental studies. No data are currently available on the diverse clinical use cases of AI for spiritual care, and empirical data are needed to clarify how the professional community evaluates AI use in clinical settings. Accordingly, this study asks how experts assess the current and future role of AI in professional spiritual care across task support and substitution, perceived risks and benefits, ethical and theological concerns, appropriate limits, required professional competencies, and whether these assessments vary across professional and demographic subgroups.

## Methods

### Ethical Considerations

Ethics approval was granted by the Ethics Committee of the Faculty of Theology and the Study of Religion, University of Zurich. Participants were required to provide informed consent prior to the survey. Participants were assigned anonymized identifiers to allow for structured feedback and incentive distribution; those who agreed to be named are listed in Multimedia Appendix 1. Upon completion of both rounds, panelists received a US $50 retail voucher.

### Study Design

**Figure 1:**
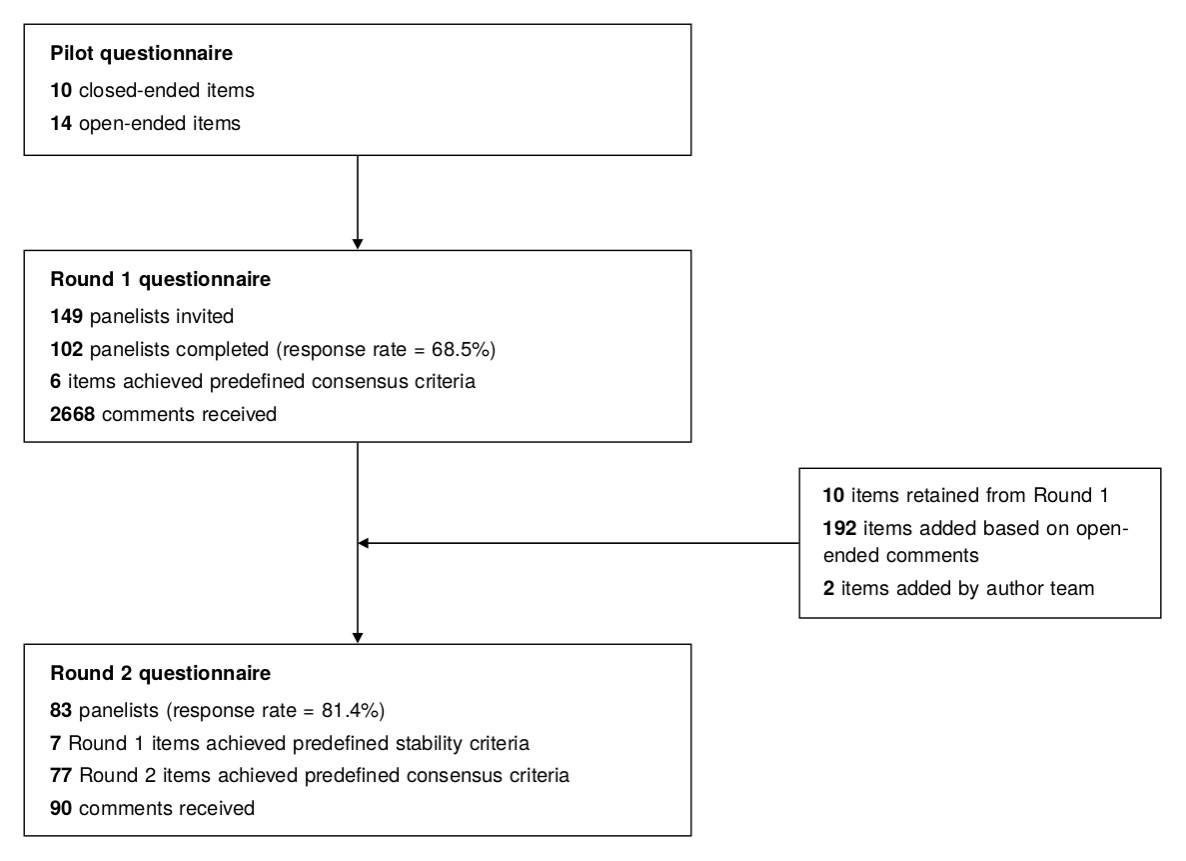
Flow diagram of the Delphi process.

The Delphi method is a technique for identifying group consensus using sequential, iterative rounds of anonymous, controlled feedback by subject experts and statistical aggregation [35]. It is an established means to identify professional consensus on clinical guidelines [36] and has been used by spiritual care researchers for diverse purposes, including the identification of research priorities [37], the development of clinical indications for interprofessional referral [38], and the advancement of curriculum standards for health care chaplaincy education [39]. Originally developed to forecast emerging trends in domains where preexisting information is incomplete or uncertain [40], the Delphi method is particularly suitable for investigating AI use in clinical practice and education [41-43]. This study employed a 2-round modified Delphi design conducted by a team of researchers in digital spiritual care, spiritual care education, and human-computer interaction (Figure 1). The team was chaired by the first author. The study is reported following the Accurate Consensus Reporting Document (ACCORD) guideline [44]. The protocol was not registered.

### Definition of Consensus

Consensus criteria (Table 1) combined measures of variance and directionality to distinguish between strength of agreement and consensus [45,46]. “Do not know” was treated as missing data to ensure that the reported consensus reflected informed sentiment. Strong agreement and strong disagreement were treated as consensus in the corresponding direction. For ranked items, consensus classifications indicated relative priority. The stopping criteria were defined as either ≥70% of items rerated in round 2 achieving stability, defined as a Wilcoxon signed-rank test *P*>.05 [47], or the response rate dropping below 70% [48].

**Table 1:** Consensus criteria for Likert-rated and ranked items.

Consensus	Likert-rated items	Ranked items
Strong agreement or high priority	Median ∈ {3, 4}; IQR≤1; Agreement: ≥80%	Weighted score in top 20th percentile; SD<1.5
Moderate agreement or moderate priority	Median ∈ {3, 4}; IQR≤1; Agreement: 60%-79%	Weighted score ≥ median; SD<1.5
Moderate disagreement or low priority	Median ∈ {1, 2}; IQR≤1; Disagreement: 60%-79%	Weighted score between 20th and 50th percentile; SD<1.5
Strong disagreement or lowest priority	Median ∈ {1, 2}; IQR≤1; Disagreement: ≥ 80%	Weighted score in bottom 20th percentile; SD<1.5
No consensus	IQR>1; or neither agreement nor disagreement: ≥60%	SD≥1.5

### Panel Selection and Recruitment

A purposive sample of subject experts was recruited for round 1 between May and September 2025 by the chair via professional networks and snowball sampling. To ensure a comprehensive multidisciplinary perspective, eligibility was restricted to board-certified (or equivalent) spiritual care providers employed at a medium or large health care organization; managers, supervisors, and directors of spiritual care departments at a medium or large health care organization; academic leaders in the research, practice, and education of spiritual care; experts in the use of AI in religious and spiritual care contexts; and experts in the ethics and theology of technology use in religious contexts. A total of 149 invitations were distributed, with approximately 30 sent to each expert category. Up to 5 reminders were sent. The recruitment target aimed for a final panel of 60-80 participants, assuming a retention rate of greater than 70% per round required for reproducible results [48].

### Data Collection (Round 1)

Data collection for round 1 was conducted between May and September 2025 with the objective of gathering broad expert consensus and generating items for subsequent assessment. To ensure a shared conceptual framework among the panel, the following definitions were provided: *“*Artificial intelligence (AI) here refers to the ability of a digital computer or computer-controlled robot to perform tasks commonly associated with intelligent beings. The term is frequently applied to the project of developing systems endowed with the intellectual processes characteristic of humans, such as the ability to reason, discover meaning, generalize, or learn from past experience” [49]. This definition was used because it is accessible to a nontechnical audience and applicable across a wide range of emerging technologies capable of performing tasks formerly dependent on human intelligence, including nongenerative (discriminative) AI used in classification or decision-making tasks such as sentiment analysis [50]. Spiritual care was defined according to long-standing professional consensus as referring to “both specialist providers (such as health care chaplains) and generalist providers (such as doctors, nurses, or social workers trained in basic spiritual support)” [51]. The initial survey included 10 Likert-rated items and 14 open-ended questions derived from an initial review of the secondary literature. The items covered 5 domains: (1) the current and potential future capabilities of AI to assist with or enhance, or substitute (ie, perform independently), specific tasks in spiritual care; (2) perceived risks and benefits; (3) theological and ethical considerations; (4) appropriate limitations to AI use; and (5) competencies needed for care providers to use AI safely and effectively in clinical settings. To minimize central tendency bias, a 4-point forced-choice Likert scale (1=strongly disagree, 2=disagree, 3=agree, and 4=strongly agree) was used, with an additional “Do not know” option included to capture uncertainty without skewing consensus. The questions were either neutral or balanced with regard to potentially negative or positive aspects of AI. Participants were prompted to explain their responses in open-ended questions or suggest changes to specific items. To avoid sequence effects, the order of Likert items was randomized for each participant. Open ranking tasks (“name and rank”) regarding expected risks, benefits, and professional competencies were included, as well as a single-choice task assessing perceived risk-benefit trade-off. The questionnaire was piloted with a convenience sample of participants to confirm face validity. Because piloting resulted only in minor wording changes and no substantive changes to item content or response structure, pilot responses were retained in the round 1 dataset.

### Data Collection (Round 2)

Data collection for round 2 was conducted between September 2025 and January 2026 to assess the stability of round 1 Likert-rated items, evaluate new items derived from initial qualitative feedback, and allow panelists to refine specific statements. Round 2 items were generated by coding the open-ended responses from round 1. The first author (FW) initially developed a coding framework by inductively coding 20% of the responses using ATLAS.ti (version 25; ATLAS.ti Scientific Software Development GmbH) [52]. No formal minimum frequency threshold was applied; codes were assigned if they captured a discrete and identifiable concept. To ensure reliability, 2 additional authors (CES and SN) independently applied this framework to the same subset [53,54]. Inclusion and consolidation of codes were decided by consensus of the author team. Overlapping codes were merged, and partially overlapping codes were retained as distinct items so the panel could differentiate related concepts within and across domains. For example, “administrative tasks” (domain Benefits and Capabilities) was kept separate to distinguish between the perception that AI systems are capable of assisting with or substituting administrative tasks and its perceived benefit. Interrater reliability for applying the final nominal codebook to the 20% validation subset reached a Krippendorff α=.792 across 3 coders, indicating acceptable intercoder reliability [55]. Following validation, the first author coded the full dataset using the coding framework. Coded text segments were then converted into discrete survey items. For example, responses to the round 1 question on current capabilities (eg, “In your view, are there currently spiritual care-related tasks that AI can substitute? Which tasks? Please list and describe them”) yielded 14 distinct task types. Similarly, free-text responses to the round 1 ranking question on expected benefits (eg, “Please name and rank the top expected benefits of AI in spiritual care”) were coded into 28 distinct themes (eg, “improved screening and triage,” “better summaries of patient data,” and “increased innovation”), which were then presented as closed-ended ranking items in round 2. The round 2 questionnaire was reviewed by the author team and the AI and Spiritual Care Special Interest Group, who suggested additional items and changes to increase face and content validity. The resulting questionnaire included 213 items.

Panelists were then invited to complete the round 2 questionnaire. First, participants were presented with their round 1 ratings, followed by summary statistics for the full panel and a representative sample of qualitative comments covering a wide range of opinions. Panelists were then asked to rate the round 2 items. After each section, they were invited to rerate round 1 items in light of the group feedback, with instructions that they could either change or maintain their initial ratings. In case of disagreement with an item, participants were asked to explain what changes to the wording of an item would be necessary to facilitate agreement. Data collection was terminated in January 2026 after reaching the predefined stopping criteria.

### Analysis

Data analysis was conducted in Python (version 3.10) using pandas, numpy, and scipy.stats, with analysis scripts generated by Claude Cowork (Opus 4.7) under full human supervision (refer to the Acknowledgments section). For Likert-rated items, median, IQR, and percent agreement were calculated. Percent agreement was defined as the proportion of nonmissing responses selecting “agree” or “strongly agree”; “do not know” responses were treated as missing. For ranked items, rank 1 was assigned 5 points, and rank 5 was assigned 1 point; items not included in a participant’s top 5 received 0 points. Weighted scores were calculated by summing these values across respondents. For items with ≥5% “do not know” responses, robustness of the consensus classification was calculated as the largest proportion of those responses that could be recoded at the opposite pole without changing the consensus classification. Panel attrition was assessed by comparing round 1 Likert ratings between returners (n=83) and dropouts (n=19) using Mann-Whitney *U* tests with rank-biserial *r* as the effect size [56]. Demographic associations were assessed using Fisher exact tests for 2 × 2 tables and chi-square tests for larger tables, with categories with n<5 excluded. Chi-square tests with expected cell counts below 5 were interpreted cautiously. To summarize whether dispersion differed across Likert-rated domains, an item-level Kruskal-Wallis test was applied to per-item standard deviations, with each item treated as one observation. Exploratory subgroup analyses tested whether ratings differed across professional and demographic groups. Mann-Whitney *U* tests were applied to all testable items within each comparison. Bonferroni familywise correction at α=.05 [57] and Benjamini-Hochberg false discovery rate (FDR) correction at q=.05 were applied within each contrast [58]. Findings were interpreted as robust if they survived at least the FDR threshold. For ranked items, subgroup differences were assessed using permutation tests on weighted-score differences. Round-to-round stability was assessed with Wilcoxon signed-rank tests on the 10 round 1 Likert items rerated in round 2, paired within round 2 returners. For the purposes of the prespecified stopping criterion, stability was defined as no statistically detectable shift at the uncorrected threshold of *P*>.05 [47].

## Results

### Results (Round 1)

Round 1 was completed by 102 of 149 invited participants (response rate: 68.5%). Median time to completion was 36 (IQR 24-60) minutes. Demographic characteristics of panelists across both rounds are reported in Table 2 and Figure S2 in Multimedia Appendix 2. Six of the 10 Likert items reached strong agreement in round 1, all in the agreement direction. Strong consensus emerged that human connection (89/100, 89%) and consciousness (81/92, 88%) are required in spiritual care, that limits on AI use are needed (84/93, 90.3%), that AI’s role is to assist with rather than substitute for spiritual care tasks (current assist: 82/97, 84.5%; future assist: 88/95, 92.6%), and that AI may, in the future, contribute to providers’ understanding of human suffering, mortality, or sacredness (84/96, 87.5%). The risk-benefit trade-off (57/76, 75%) and the current-understanding item (73/95, 76.8%) reached only moderate agreement, while the current and future substitution items (46/92, 50% and 48/89, 53.9%) did not reach consensus. Per-item descriptive statistics are reported in Table 3 as well as Table S5 in Multimedia Appendix 3. Free-text responses to the 14 open-ended questions yielded 2668 comments, organized into 172 codes in 6 domains (task types: 41; benefits: 33; risks: 34; limits: 25; theological or ethical: 22; competencies: 17), plus 3 administrative codes for nonresponses. These codes were operationalized into the round 2 closed-ended items using the procedure described in the Methods section.

**Table 2:** Demographic characteristics of panelists in each round of the Delphi survey.

Characteristic		Round 1	Round 2
Invited, n		149	102
Completed, n		102	83
Response rate, %		68.5	81.4
**Gender, n (%)**			
	Male	57 (55.9)	49 (59)
	Female	44 (43.1)	33 (39.8)
	Non-binary	1 (1)	1 (1.2)
**Age (years), n (%)**			
	30-39	22 (21.6)	17 (20.5)
	40-49	30 (29.4)	23 (27.7)
	50-59	21 (20.6)	19 (22.9)
	60-64	16 (15.7)	12 (14.5)
	≥65	13 (12.7)	12 (14.5)
**Religious identification, n (%)**			
	Mainline Protestant	42 (41.2)	35 (42.2)
	Catholic	12 (11.8)	7 (8.4)
	Evangelical	7 (6.9)	7 (8.4)
	Humanist	5 (4.9)	2 (2.4)
	Other Christian	5 (4.9)	4 (4.8)
	Jewish	4 (3.9)	4 (4.8)
	Buddhist	3 (2.9)	3 (3.6)
	Muslim	3 (2.9)	3 (3.6)
	No religious identification	1 (1)	1 (1.2)
	Spiritual but not religious	1 (1)	1 (1.2)
	Not reported	19 (18.6)	16 (19.3)
**Professional background, n (%)**			
	Spiritual care and chaplaincy	61 (59.8)	51 (61.4)
	Theology and religious studies	20 (19.6)	16 (19.3)
	Medicine	7 (6.9)	5 (6)
	Ethics	4 (3.9)	2 (2.4)
	Computer science and IT	3 (2.9)	3 (3.6)
	Other	7 (6.9)	6 (7.2)
**Employer, n (%)**			
	Hospital or health system	49 (48)	39 (47)
	University	40 (39.2)	31 (37.3)
	Nonprofit organization	6 (5.9)	6 (7.2)
	For-profit organization	1 (1)	1 (1.2)
	Governmental organization	1 (1)	1 (1.2)
	Private health practice	1 (1)	1 (1.2)
	Other	4 (3.9)	4 (4.8)
**Title or position, n (%)**			
	Board-certified chaplain	30 (29.4)	22 (26.5)
	Professor	20 (19.6)	13 (15.7)
	Head of department	12 (11.8)	10 (12)
	Postdoctoral or other researcher	9 (8.8)	7 (8.4)
	Assistant professor	7 (6.9)	7 (8.4)
	Other spiritual care provider	5 (4.9)	5 (6)
	President, vice-president, or equivalent	5 (4.9)	5 (6)
	Associate professor	4 (3.9)	4 (4.8)
	Program director	4 (3.9)	4 (4.8)
	Clinician (eg, physician or nurse)	1 (1)	1 (1.2)
	Other	5 (4.9)	5 (6)
**Professional activities, n (%)**			
	Research	35 (34.3)	26 (31.3)
	Clinical	27 (26.5)	19 (22.9)
	Management	22 (21.6)	20 (24.1)
	Teaching	14 (13.7)	14 (16.9)
	Other	4 (3.9)	4 (4.8)
**Location, n (%)**			
	North America	67 (65.7)	54 (65.1)
	Europe	32 (31.4)	26 (31.3)
	Asia	2 (2)	2 (2.4)
	Oceania	1 (1)	1 (1.2)

**Table 3:** Round 1 Likert and open-ended questions, in order of percentage agreement. The full table of Round 1 results is in Multimedia Appendix 3.

Item	Frequency, n	Median (IQR)^a^	Agreement, %	Consensus	Stable	Open-ended questions
Do you expect that, in the future, AI might be able to assist with or enhance specific tasks in human-provided spiritual care?	95	3 (1)	92.6	Strong agreement	N^b^	Which tasks? Please list and describe them.
Should there be limits placed on what types of spiritual care AI can provide?	93	4 (1)	90.3	Strong agreement	Y^c^	If yes, what kinds of limits? Please name and rank the professional competencies spiritual care providers would need to safely and effectively use AI in patient care. Rank from 1 (most important) to 10 (least important).
Should AI-provided spiritual care always involve or connect back to a human caregiver?	100	4 (1)	89	Strong agreement	Y	Which spiritual care-related tasks should always involve or connect back to a human caregiver?
Do you believe effective spiritual care requires the care provider to have human consciousness?	92	4 (1)	88	Strong agreement	Y	Which spiritual care-related tasks require the care provider to have human consciousness?
Do you expect that, in the future, AI might be able to contribute to a spiritual care provider’s understanding of human suffering, mortality, or sacredness?	96	3 (1)	87.5	Strong agreement	Y	If you agree, how?
Is AI currently able to assist with or enhance specific tasks in human-provided spiritual care?	97	3 (0)	84.5	Strong agreement	Y	Which tasks? Please list and describe them.
In your view, is AI currently able to contribute to a spiritual care provider’s understanding of human suffering, mortality, or sacredness?	95	3 (0)	76.8	Moderate agreement	Y	If you agree, how?
In your view, do the potential benefits of AI in spiritual care outweigh the potential risks?	76	3 (1)	75	Moderate agreement	Y	Please explain with reference to the potential risks and benefits you mentioned. Please name and rank the potential benefits of AI use by spiritual care providers from 1 (most important) to 10 (least important). Please name and rank the potential risks of AI use by spiritual care providers from 1 (highest risk) to 10 (lowest risk).
Do you expect that, in the future, there may be some spiritual care-related tasks which AI might be able to substitute?	89	3 (1)	53.9	No consensus	N	Which tasks? Please list and describe them.
In your view, are there currently spiritual care-related tasks that AI can substitute?	92	2.5 (1)	50	No consensus	N	Which tasks? Please list and describe them.

^a^Likert items were assessed using a 4-point ordinal scale. Consensus criteria were defined based on interquartile width; the IQR is reported as an integer width for Likert items.

^b^N indicates unstable (Wilcoxon signed-rank test *P*≤.05).

^c^Y indicates stable (Wilcoxon signed-rank test *P*>.05).

### Results (Round 2)

Of the 102 round 1 panelists invited to round 2, a total of 83 completed the second survey (response rate: 81.4%). Median time to completion was 34 (IQR 28-76) minutes. Seven of the 10 rerated items met the stability criterion. The three items that shifted between rounds all moved toward greater acceptance of AI’s role in spiritual care tasks; the largest shift was on the round 1 Likert item on future task substitution (“Do you expect that, in the future, there may be some spiritual care-related tasks which AI might be able to substitute?”), which moved from no consensus in round 1 (53.9% agree) to strong agreement in round 2 (80% agree; Wilcoxon *P*<.001); the median remained at 3, but 52.2% of paired panelists revised their rating between rounds. Across all 10 items, an average of 38.7% of panelists changed their rating between rounds (range 25.5%-52.7%). The strongest current-assistance items were administrative and routine tasks (77/81, 95.1%), informational tasks (74/79, 93.7%), documentation (67/80, 83.8%), and spiritual care research (65/77, 84.4%). In contrast, agreement was lower for tasks involving direct relational or ritual dimensions of care, including creating supportive spaces (30/69, 43.5%), direct patient engagement (32/76, 42.1%), and conducting ritual tasks (32/76, 42.1%). This indicates that panelists distinguished between AI as a support for informational, administrative, and documentation work and AI involvement in activities closer to the relational aspects of spiritual care. A summary of per-item descriptive statistics is reported in Table 4, with full results in Tables S6-S10 in Multimedia Appendix 4.

Agreement varied across the 3 Likert-rated domains: the limits domain showed the greatest convergence, with 75% of items reaching strong agreement, while the tasks and theological and ethical domains were similar at 46% and 42%, respectively. An item-level Kruskal-Wallis test on per-item standard deviations indicated that response dispersion differed across domains (H=20.19, *df*=2; *P*<.001; η²_H=.15), consistent with tighter convergence in the limits domain. The same pattern appeared at the item level: the panel tended to converge on professional limits but diverge on theological and ethical objections.

Among the 85 items with ≥5% “do not know” responses, 55 reached one of the predefined consensus classifications. For these items, the median robustness was 87.5% (IQR 35.9%-100%), suggesting that most consensus classifications were not highly sensitive to the exclusion of “do not know” responses.

Subgroup analyses produced few robust differences. Region was the only contrast with findings that survived both Bonferroni and FDR correction: European respondents ranked “engage in self-reflection on how AI use affects care practice” more highly as a competency (*P*=.001), while North American respondents ranked “improved screening and triage” more highly as a benefit (*P*=.001).

None of the 10 round 1 Likert items showed significant differences between returners and dropouts (smallest *P*=.06 for “Human connection required for spiritual care”). All effect sizes were small (|r_rb|≤.24), and directions across items were mixed, inconsistent with systematic attrition bias. Items most relevant to plausible attrition biases were tested directly: neither the risk-benefit trade-off (*P*=.52) nor the human-consciousness requirement (*P*=.40) showed a detectable difference between groups. None of the demographic variables tested showed a significant association with returner status (smallest *P*=.06 for primary professional activity, not surviving correction).

**Table 4:** Summary of round 2 consensus (N=83). For Likert-rated items, one representative item is shown per consensus category present in each subsection: the highest-ranked strong agreement item, the median moderate agreement item, and the lowest-ranked disagreement item. For ranked items, the 3 highest-ranked high-priority items are shown. The full table of Round 2 results is in Multimedia Appendix 4.

Domain and item				Frequency, n		Median (IQR)		Agreement, %		Classification
**AI capabilities**										
	**AI is able to contribute to a spiritual care provider’s understanding of human suffering, mortality, or sacredness in the following ways (present)**									
		Support providers’ understanding but cannot replace human wisdom, empathy, or discernment	81		4 (1)		92.6		Strong agreement	
		Generate creative works that shed light on these themes	76		3 (0)		78.9		Moderate agreement	
		AI cannot contribute to understanding of suffering, mortality, or sacredness	79		2 (0)		19		Strong disagreement	
	**AI is able to contribute to a spiritual care provider’s understanding of human suffering, mortality, or sacredness in the following ways (future)**									
		Reveal patterns in patient narratives of suffering, mortality, and sacredness	79		4 (1)		97.5		Strong agreement	
		AI cannot contribute to understanding of suffering, mortality, or sacredness	75		2 (1)		18.7		Strong disagreement	
	**AI is able to assist with or enhance the following tasks (present)**									
		Administrative and routine tasks (e.g., schedule patients, recommend follow-ups, produce reports)	81		3 (1)		95.1		Strong agreement	
		Create legacy tasks and advance directives (e.g., write ethical wills, life reviews, or farewell letters based on patient inputs)	76		3 (1)		73.7		Moderate agreement	
	**AI is able to assist with or enhance the following tasks (future)**									
		Administrative and routine tasks (eg, schedule patients, recommend follow-ups, and produce reports)	80		4 (1)		98.8		Strong agreement	
		Direct patient engagement (eg, engage patients in conversation, provide words of care and comfort, and conduct remote check-ins)	72		3 (1)		62.5		Moderate agreement	
	**AI can substitute the following tasks (present)**									
		Conduct research in spiritual care (eg, analyze encounter data, identify trends, and produce summaries for studies)	79		3 (1)		73.4		Moderate agreement	
		Create supportive spaces (eg, host peer-support groups, moderate online communities, and create immersive virtual prayer and meditation environments)	74		2 (1)		18.9		Strong disagreement	
	**AI can substitute the following tasks (future)**									
		Administrative and routine tasks (eg, schedule patients, recommend follow-ups, and produce reports)	79		4 (1)		94.9		Strong agreement	
		Train spiritual care providers (eg, role-play case scenarios, simulate empathic responses, and generate case studies)	75		3 (1)		70.7		Moderate agreement	
		Conduct ritual tasks (eg, guide meditations, recite or adapt prayers, and lead ceremonies)	76		2 (1)		32.9		Moderate disagreement	
**Risks and benefits^a^**										
	**The potential benefits of AI use by spiritual care providers are**									
		Improved screening and triage (108)	31		—^b^		—		High priority	
		Improved documentation (107)	38		—		—		High priority	
		Better access to information (99)	35		—		—		High priority	
	**The potential risks of AI use by spiritual care providers are**									
		Loss of human connection (122)	39		—		—		High priority	
		Dehumanization of spiritual care (117)	34		—		—		High priority	
		Uncritical use of AI (103)	28		—		—		High priority	
**Risk-benefit trade-off^a^**										
	**In your view, do the potential benefits of AI in spiritual care outweigh the potential risks?**									
		The benefits outweigh the risks	38		—		45.8		—	
		The benefits and risks are about equal	18		—		21.7		—	
		Do not know or it depends	15		—		18.1		—	
		The risks outweigh the benefits	12		—		14.5		—	
**Theological and ethical considerations**										
	**Do you believe effective spiritual care requires the care provider to have human consciousness?**									
		Human presence gives spiritual care a moral, spiritual, or emotional depth that AI cannot replicate	78		4 (1)		94.9		Strong agreement	
		Spiritual care should be judged by its benefit to the patient, not by whether the caregiver is human	73		3 (1)		60.3		Moderate agreement	
	**Should AI-provided spiritual care always involve or connect back to a human caregiver?**									
		AI should only be used as a support tool, with final responsibility resting with a human	79		4 (1)		94.9		Strong agreement	
		AI may provide care independently for simple or routine tasks	77		3 (1)		71.4		Moderate agreement	
	**The following theological or ethical objections to the use of AI in spiritual care should be considered**									
		Substituting AI for embodied human presence undermines the sacred dimension of care	73		3 (1)		86.3		Strong agreement	
		AI use in spiritual care raises theological concerns about creation, Imago Dei, or human uniqueness	76		3 (1)		68.4		Moderate agreement	
		AI use in spiritual care brings serious harm, including spiritual trauma	66		2 (1)		37.9		Moderate disagreement	
	**The following limits should be placed on the use of AI in spiritual care**									
		Implementation of AI in spiritual care must be based on evidence of safety and effectiveness	82		4 (1)		100		Strong agreement	
		AI-enabled chatbots must be customized to reflect the patient’s faith or spiritual tradition	68		3 (1)		75		Moderate agreement	
		No limits or requirements are needed or possible	80		1 (0)		6.3		Strong disagreement	
**Professional competencies^c^**										
	**Spiritual care providers would need the following professional competencies to safely and effectively use AI in patient care**									
		Judge when AI is applicable and identify boundaries of its use (153)	49		—		—		High priority	
		Safeguard patient privacy and data in AI contexts (124)	37		—		—		High priority	
		Assess and mitigate risks associated with AI use (119)	35		—		—		High priority	

^a^Single-choice categorical question; percentages are calculated over total respondents.

^b^Not applicable.

^c^Ranked items: parenthetical values are weighted priority scores (rank 1=5 points; rank 5=1 point); n=panelists selecting item in their top 5.

## Discussion

### Principal Findings

#### Administrative, Informational, and Research Tasks

The panel’s strongest agreement was on the ability of AI to engage in administrative and informational tasks such as documentation and research. Agreement was strongest for AI assistance with these tasks, while substitution was more task dependent. The panel exhibited moderate to strong agreement that some administrative, informational, documentation, and research-related tasks could be substituted by AI in the present or future. While broadly consistent with the general trend toward the automation of routine informational labor, this sentiment warrants further consideration in light of the tendency of generative AI systems used in health care to hallucinate outputs [59,60] and the potentially far-reaching consequences of AI-related errors in clinical settings, even in administrative and informational tasks. The consensus regarding substitution in these domains suggests a need for closer examination of how panelists envisioned implementation in practice.

Agreement about AI use in documentation, for example, may reflect assumptions about ambient AI scribes and similar automated transcription and charting technologies, which may differ in autonomy, patient proximity, and risk profile. For instance, a tool developed by Epic Systems [61] can format chart notes as bullet points and record patient-provider conversations to draft notes and suggest clinician orders. While the former may be perceived as representing a relatively unproblematic case of task assistance, automated charting by “AI scribes” based on voice-recorded patient-provider interactions is error-prone [62,63]. Moreover, while some clinicians report reduced cognitive load and more time to interact with patients [64], critics warn against the risk of providers performing for the microphone instead of tending to the interpersonal aspects of therapeutic communication [65]. Professional discernment, guidance, and AI literacy training may be needed to identify AI implementations that are appropriate to professional practice and preserve the relational aspect of patient care central to the profession.

#### Professional Discernment and Caution

Panelists also perceived AI as capable of assisting with tasks such as clinical decision-making and prioritization, which, while often routine and informational, require considerable human discernment. The panelists also expressed moderate to strong agreement regarding the ability of AI to assist with or enhance legacy tasks and advance directives, which involve ethical complexity and patient vulnerability. Perhaps due to the intermingling of administrative and relational tasks in clinical practice, the panel expressed strong agreement that spiritual care providers should be competent to “judge when AI is applicable and identify boundaries of its use.” This was the highest-ranked professional competency needed by providers to safely and effectively use AI in patient care.

The highest-ranked risks—loss of human connection, dehumanization of spiritual care, and uncritical use of AI—suggest that panelists were concerned not only with technical safety but also with how AI use might affect the human practice of care. This is consistent with recent findings that currently available chatbots configured to imitate conversational features of human spiritual care providers are unable to replicate the presence conveyed by human providers [66], and AI systems exhibit deceptive empathy and poor therapeutic collaboration even when prompted to follow evidence-based psychotherapeutic techniques [67]. A scoping review of stakeholder attitudes toward AI in clinical practice similarly identified a potential trade-off between greater efficiency and less patient-centered care [68]. Resistance in this domain may reflect a deeper professional commitment to preserving the relational core of spiritual care providers as domain experts in human care.

#### Future Orientation and Conditional Acceptance

Panelists agreed more strongly on the future abilities of AI than on its current ability to assist, enhance, or substitute for tasks. This may indicate that current skepticism reflects perceived limitations of contemporary AI technologies rather than the view that AI is categorically incapable of performing certain tasks. The panel’s optimism is consistent with public sentiments regarding the future transformative potential of AI technologies. However, items involving relational tasks showed less movement between rounds and across temporal framings: panelists remained comparatively cautious about AI capabilities in direct patient engagement, supportive presence, and ritual practice. This suggests that the boundary around relational care is not simply a response to current technical limitations but may reflect a more stable professional judgment about which aspects of spiritual care will continue to require human involvement, even as AI capabilities continue to develop in the future.

Although nearly half of panelists perceived the potential benefits of AI in spiritual care to outweigh the potential risks, they strongly converged on several competencies required for safe and effective AI use, including judging applicability and boundaries, safeguarding patient privacy and data, and assessing and mitigating risks. These attitudes may not remain static: a recent population survey found that acceptance of digital tools in spiritual care contexts is significantly related to age and previous experience, suggesting that attitudes may evolve as both patients and providers become increasingly accustomed to AI use in daily life [69]. Related research on digital spiritual care also suggests that adoption is shaped by institutional and organizational conditions, indicating that acceptability is context dependent rather than fixed [70,71]. Longitudinal and implementation-focused research will be needed to understand how professional consensus develops alongside AI capabilities and institutional norms.

#### Convergence on Practice, Divergence on Principle

The panel converged on professional limits for AI use but diverged on the theological and ethical objections that justify those limits. For example, the limit “AI must not perform sacraments or lead rituals” reached strong agreement (86.8%, IQR 1), while the parallel objection (“Religious rituals and sacraments performed by AI are invalid or inappropriate”) did not reach consensus (70.8%, IQR 2). A similar gap appeared for the role of AI in ethical and moral decision-making: as a limit on professional practice, this reached near-universal agreement (96.3%, IQR 1), but framed theologically—that such decisions should rest with religious authorities—it did not reach consensus (40.8%, IQR 2). This pattern may be argued to represent what Cass R. Sunstein refers to as “incompletely theorized agreement,” in which agreement exists on what should be done despite disagreement or uncertainty regarding the underlying principles of that agreement [72]. This could be interpreted to imply that the professional consensus is superficial and potentially fragile. However, as Sunstein suggests, “avoidance of more abstract or theoretical claims” may be a pragmatic or strategic choice or aim to circumvent “highly abstract questions” that are perceived to be “too hard, large, and open-ended [...] to handle” [72]. Incompletely theorized agreement may be a necessary feature of the spiritual care community, which has established widely accepted professional boundaries, such as the commitment to nonproselytization and an international consensus definition of “spirituality” [73], despite substantial regional, demographic, and worldview differences among providers. The present findings suggest that AI guidance could develop similarly: professional standards may specify a baseline agreement on red lines and acceptable use cases, even if practitioners disagree about the theological or ethical reasons for those limits.

#### Consensus Across Subgroups

The absence of corrected subgroup differences suggests that professional opinion was not strongly shaped by the demographic, religious, professional, and other contextual factors measured in this study. In particular, exploratory analyses comparing chaplaincy-trained respondents with other panelists did not produce corrected item-level differences. Exploratory analyses by religious affiliation likewise did not identify corrected differences, although the small number of non-Christian respondents means that tradition-specific variation cannot be ruled out and should be examined in future work with religiously stratified samples.

Region was the only subgroup dimension with corrected differences. The higher ranking of “engage in self-reflection on how AI use affects care practice” as a competency among European respondents is consistent with a more risk-based orientation toward technological innovation and AI governance, as reflected in recent European regulatory developments [74]. The higher ranking of “improved screening and triage” as a benefit among North American respondents may reflect the greater professionalization and routine use of screening and triage within North American professional practice. However, because no consistent pattern of regional or other subgroup divergence appeared across the remaining items, these differences should be interpreted cautiously. Overall, the findings suggest that professional opinion on AI in spiritual care was broadly shared across the panel.

### Recommendations

Spiritual care providers may need to weigh AI use and nonuse in relation to patient needs, rapidly advancing technologies, and evolving theological, organizational, and professional standards. Research in the field of human-AI interaction has shown the importance of understanding how AI systems can support clinical practice to ensure they do not disrupt workflows, increase staff burden, fail to align with local routines, or adversely affect clinician trust [75,76]. Spiritual care educators and professional associations may wish to consider creating detailed guidelines on how tasks, control, and responsibility are allocated, including when AI systems may act, when they should defer, and how providers verify, correct, or override outputs [77,78]. The expert opinions mapped here point toward areas where future guideline development could focus, in particular, strong safeguards and principled professional practices regarding human responsibility, patient privacy, bias, consent, and the ethical and theological boundaries of AI use. New design frameworks for AI-enabled technologies and guidance by professional organizations may help operationalize these recommendations [79]. Finally, the breadth of use cases for which experts saw some role for AI may suggest a need to broaden the current interest in chatbots in patient care and conduct empirical research based on a task-differentiated framework. Future research may wish to focus on patient perspectives, specific AI applications, clinical settings, and religious traditions. Task-differentiated outcome studies would be needed to understand the implications of AI use, particularly in direct patient care. Finally, the professional community may wish to develop competency models that preserve the relational and ethical commitments central to spiritual care in AI-enabled clinical workflows.

### Limitations

The study design was restricted to professional consensus and did not include patient perspectives, needs, and preferences. The panel was geographically concentrated in North America and Europe and weighted toward spiritual care and chaplaincy expertise. Although some panelists had substantial expertise in the ethics and theology of AI, others were primarily highly experienced spiritual care professionals. Because the study did not assess participants’ level of AI literacy, findings should be interpreted as expert judgment shaped by varying degrees of technical familiarity with AI. Due to the lack of available secondary literature during the study planning phase, the initial questionnaire focused on broad questions. To manage participants’ cognitive load, the complexity of the questionnaire was reduced. Consequently, the study did not investigate additional context-specific factors; did not differentiate between different worldviews and religious traditions or between specific AI products and clinical settings; and aggregated task-specific risk-benefit trade-offs, limitations, and ethical and theological considerations. The practical details of human supervision of AI activities were also beyond the scope of this study. Three members of the author team participated as panelists. Consensus among experts is a form of evidence with known constraints. Due to these limitations, this study represents an exploratory map of current expert opinions.

### Conclusions

This study provides the first task-level map of expert opinion on AI use in spiritual care. The findings suggest that current expert opinions in spiritual care are task specific, future oriented, and conditional. AI is generally viewed as useful for administrative, informational, documentation, and research-related tasks, while relational tasks remain widely understood as a primarily human responsibility. However, some AI use cases with moderate to strong agreement are ethically complex and require further consideration. Experts also identified clear risks, especially loss of human connection, dehumanization of spiritual care, and uncritical use of AI. Strong convergence appeared around practical safeguards, including human oversight, evidence-based implementation, informed use, consent, privacy protection, and clear limits on AI decision-making. The lack of substantial subgroup differences and convergence on specific limitations suggests that professional guidance may be possible even where deeper theological or ethical rationales remain contested.

In sum, this study identifies areas of emerging professional convergence and disagreement at an early stage of AI adoption in spiritual care and may inform future research, professional guidance, and curriculum development.

## Appendix

Multimedia Appendix 1 List of panelists.

Multimedia Appendix 2 Bar charts of demographic characteristics.

Multimedia Appendix 3 Results (round 1).

Multimedia Appendix 4 Results (round 2).

## Abbreviations

ACCORD: Accurate Consensus Reporting Document
APC: Association of Professional Chaplains
ETHOS: Ethical Technology for Human Oriented Spiritual Care
FDR: false discovery rate

## Data Availability

The datasets generated or analyzed during this study are not publicly available in accordance with the terms of the institutional ethics approval.
