# Supplementary material for: Artificial Intelligence in Spiritual Care: Modified Delphi Study
Source: J Med Internet Res. 2026 Jul 27;28:e97094. doi: 10.2196/97094 (PMC13458354; doi:10.2196/97094)
Supplement: Supplementary file 1 [file jmir_v28i1e97094_app1.pdf]

Appendix 4: List of panelists

Aaron Unseth, Allina Health; Abdurrahman Anwar, Barts Health NHS; Abraham Labrada-Santiago, Mayo Clinic Rochester; AJ Gutierrez, AdventHealth; Alemitu Bezabih, Colorado School of Mines; Alex Schapiro, Atlantic Health System; Alison Kestenbaum, UC San Diego Health; Amanda Borchik, Rush University Medical Center; Amitashuri Yule, NHS Greater Glasgow and Clyde; Andrew Proudfoot, University of Nottingham; Andrew Savitz, SpirituWell; Andrew Williams, Oxford Health NHS Foundation Trust; Angelika A. Zollfrank, McLean Hospital; Anne Foerst, St. Bonaventure University; Annette Haußmann, Heidelberg University; Antonia Lüdtke, Christian-Albrecht University of Kiel; Bronwen Jones, Cedars-Sinai Medical Center; C. Estelle Smith, Colorado School of Mines; Caitlin Beaulieu-Desjardins, Rush University Medical Center; Carrie Doehring, Iliff School of Theology; Carrie Kohler, Cedars-Sinai Medical Center; Charles Ess, University of Oslo; Charlie Shin, Providence; Chris Swift, Leeds Church Institute; Christina Puchalski, George Washington University; Chuck Valenti-Hein, Ascension Health; Cody Alley, Mercy; Csaba Szilagyi, Rush University Medical Center; Curtisha Grant, Baylor Scott & White Health; Dana T. Fachner, University of Arkansas at Little Rock; Daniel Grossoehme, Akron Children's Hospital; Dan Roberts, Baylor Scott & White Health; Daniel Nuzum, Cork University Hospitals Group; Darrell Cousino, Bon Secours Mercy Health; David Fleenor, ArchCare; Dilek Uçak Ekinci, QuaMS – Muslimische Seelsorge Zürich; Eckhard Frick, Technical University of Munich; Eric Hammer, Baylor Scott & White Health; Frauke Rörden, Ev.-Luth. Kirche in Norddeutschland; Gena Maria Parker, Mayo Clinic; George Handzo, HealthCare Chaplaincy Network; Georg Lämmlin, Sozialwissenschaftliches Institut der Evangelischen Kirche in Deutschland; Greg Epstein, Harvard University; Heidi A. Campbell, Texas A&M University; Hugo Gonzales, formerly Lucile Packard Children's Hospital; Ilona Nord, University of Würzburg; Jaco Hamman, Vanderbilt University; James Adams, Houston Methodist Hospital; James Barr, Atlantic Health; Jane Jeuland, Yale New Haven Hospital; Jason Weiner, Cedars-Sinai Medical Center; Jason Wilson, The Johns Hopkins Hospital; Joana Romeira, Universidade Católica Portuguesa; John Swinton, University of Aberdeen; Jonas Simmerlein, University of Vienna; Josiah Hoagland, CommonSpirit Health; Justin Martin, Mercy; Katarina Holmgren, Penn Medicine Lancaster General Health; Kelsey White, Virginia Commonwealth University; Kevin Correll, Lehigh Valley Health Network; Lavera Crawley, CommonSpirit Health; Lindsay Carey, La Trobe University; Lindsay de Wal, Sheffield Teaching Hospitals NHS Foundation Trust; Megan Best, University of Notre Dame Australia; Michael Coors, University of Zurich; Mitchell Chilcott, formerly Providence; Niels Christian Hvidt, University of Southern Denmark; Nika Höfler, Evangelisch-Lutherische Kirche in Bayern; Pamela Cooper-White, Union Theological Seminary; Paul Galchutt, Rush University Medical Center; Pauline H. Cheong, Arizona State University; Petra Sprik, University of Alabama at Birmingham; Piret Paal, University of Tartu; Ralf-Peter Reimann, Evangelische Kirche im Rheinland; Reinhard Grabenweger, Paracelsus Medizinische Privatuniversität; Rhonda James-Jones, WellStar Health System; Ricardo Chavarriaga, Zurich University of Applied Sciences; Ricko Damberg Nissen, University of Southern Denmark; Robert E. Brown, IU Health Methodist Hospital; Robert Geraci,

Knox College; Ruth Tsuria, Seton Hall University; Sarah Diamant, New York-Presbyterian Hospital; Shelley E. Varner-Perez, Indiana University Health; Stephen King, Fred Hutchinson Cancer Center; Stephen Ott, formerly New York Presbyterian-Lawrence Hospital; Takeshi Kimura, University of Tsukuba; Terra Tindle Williams, Providence; Terri Vilagos, Advocate Health; Theresa Edmonson, Providence; Thomas Zeilinger, Friedrich-Alexander University of Erlangen–Nuremberg; Tim Hutchings, University of Nottingham; Timothy Gaura, Arnot Health; Timothy Yoder, Mayo Clinic; Trace Haythorn, formerly Association of Clinical Pastoral Education (ACPE); Tracy Balboni, Harvard Medical School; Tracy Trothen, Queen's University; Traugott Roser, University of Münster; Wim Smeets, Radboudumc; Yong Sup Song, Seoul Christian University; Yulia Kazakova, CHI Health; Zentrum für Seelsorge und Beratung, Ev.-Luth. Landeskirche Hannover and one anonymous panelist.
