# Supplementary material for: Artificial Intelligence in Spiritual Care: Modified Delphi Study
Source: J Med Internet Res. 2026 Jul 27;28:e97094. doi: 10.2196/97094 (PMC13458354; doi:10.2196/97094)
Supplement: Supplementary file 2 [file jmir_v28i1e97094_app2.pdf]

Appendix 1: Bar charts of demographic characteristics

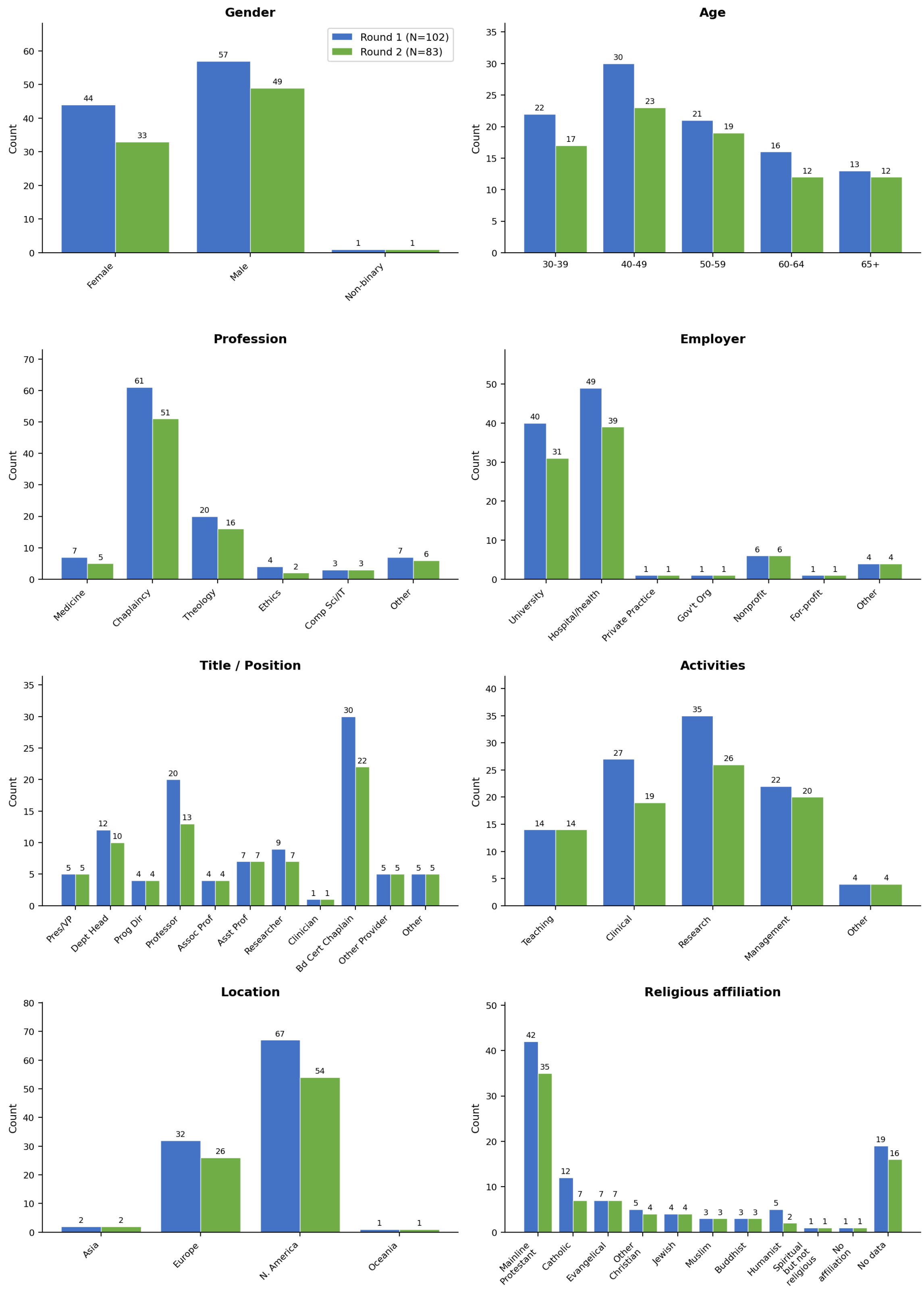

Figure S2: Bar charts of demographic characteristics
