# Supplementary material for: Artificial Intelligence in Spiritual Care: Modified Delphi Study
Source: J Med Internet Res. 2026 Jul 27;28:e97094. doi: 10.2196/97094 (PMC13458354; doi:10.2196/97094)
Supplement: Supplementary file 3 [file jmir_v28i1e97094_app3.pdf]

Appendix 2: Results (round 1)

Likert-rated questions	n	Don't Know	Mdn	IQR	Agree (%)	Consensus	Stable	Open-ended questions
Do you expect that, in the future, AI might be able to assist with or enhance specific tasks in human-provided spiritual care?	95	7	3	1	92.6	Strong agreement	No (P=.04)	Which tasks? Please list and describe them.
Should there be limits placed on what types of spiritual care AI can provide?	93	8	4	1	90.3	Strong agreement	Yes (P=.06)	- If yes, what kinds of limits?<br>- Please name and rank the professional competencies spiritual care providers would need to safely and effectively use AI in patient care. Rank from 1 (most important) to 10 (least important).
Should AI-provided spiritual care always involve or connect back to a human caregiver?	100	2	4	1	89.0	Strong agreement	Yes (P=.20)	Which spiritual care-related tasks should always involve or connect back to a human caregiver?
Do you believe effective spiritual care requires the care provider to have human consciousness?	92	10	4	1	88.0	Strong agreement	Yes (P=.12)	Which spiritual care-related tasks require the care provider to have human consciousness?
Do you expect that, in the future, AI might be able to contribute to a spiritual care provider's understanding of human suffering, mortality, or sacredness?	96	6	3	1	87.5	Strong agreement	Yes (P=.20)	If you agree, how?
Is AI currently able to assist with or enhance specific tasks in human-provided spiritual care?	97	5	3	0	84.5	Strong agreement	Yes (P=.49)	Which tasks? Please list and describe them.
In your view, is AI currently able to contribute to a spiritual care provider's understanding of human suffering, mortality, or sacredness?	95	7	3	0	76.8	Moderate agreement	Yes (P=.59)	If you agree, how?
In your view, do the potential benefits of AI in spiritual care outweigh the potential risks?	76	26	3	1	75.0	Moderate agreement	Yes (P=.99)	- Please explain with reference to the potential risks and benefits you mentioned.<br>- Please name and rank the potential benefits of AI use by spiritual care providers from 1 (most important) to 10 (least important).<br>- Please name and rank the potential risks of AI use by spiritual care providers from 1 (highest risk) to 10 (lowest risk).
Do you expect that, in the future, there may be some spiritual care-related tasks which AI might be able to substitute?	89	13	3	1	53.9	No consensus	No (P<.001)	- Which tasks? Please list and describe them.
In your view, are there currently spiritual care-related tasks that AI can substitute?	92	10	2.5	1	50.0	No consensus	No (P=.02)	Which tasks? Please list and describe them.

Table S5. Likert and open-ended questions (round 1) with consensus and stability rating based on round 2 rerate, in order of % agree.
