# Supplementary material for: Artificial Intelligence in Spiritual Care: Modified Delphi Study
Source: J Med Internet Res. 2026 Jul 27;28:e97094. doi: 10.2196/97094 (PMC13458354; doi:10.2196/97094)
Supplement: Supplementary file 4 [file jmir_v28i1e97094_app4.pdf]

Appendix 3: Results (round 2)

		Currently						In the future					
		n	Don't Know	Mdn	IQR	Agree (%)	Consensus	n	Don't Know	Mdn	IQR	Agree (%)	Consensus
AI is able to contribute to a spiritual care provider's understanding of human suffering, mortality, or sacredness in the following ways: (Likert)	Support providers' understanding but cannot replace human wisdom, empathy, or discernment	81	2	4	1	92.6	Strong agreement	76	7	4	1	94.7	Strong agreement
	Reveal patterns in patient narratives of suffering, mortality, and sacredness	77	6	3	0	88.3	Strong agreement	79	4	4	1	97.5	Strong agreement
	Provide a superficial or textbook understanding of these themes	80	3	3	1	87.5	Strong agreement	79	4	3	1	88.6	Strong agreement
	Broaden perspective on intercultural and interreligious dimensions of suffering, mortality, or sacredness	77	6	3	0	85.7	Strong agreement	76	7	3	1	92.1	Strong agreement
	Generate creative works that shed light on these themes	76	7	3	0	78.9	Moderate agreement	70	13	3	1	91.4	Strong agreement
	AI cannot contribute to understanding of suffering, mortality, or sacredness	79	4	2	0	19.0	Strong disagreement	75	8	2	1	18.7	Strong disagreeme nt
AI is able to assist with or enhance the following tasks: (Likert)	Administrative and routine tasks (e.g., schedule patients, recommend follow-ups, produce reports)	81	2	3	1	95.1	Strong agreement	80	3	4	1	98.8	Strong agreement
	Summarize and classify information (e.g., build taxonomies of spiritual needs, condense patient stories, summarize prayers or scriptures)	79	4	3	1	93.7	Strong agreement	80	3	4	1	92.5	Strong agreement
	Conduct research in spiritual care (e.g., analyze encounter data, identify trends, produce summaries for studies)	77	6	3	1	84.4	Strong agreement	78	5	4	1	92.3	Strong agreement
	Documentation and care planning (e.g., draft chart notes, generate patient profiles, pre-fill care plans)	80	3	3	0	83.8	Strong agreement	80	3	4	1	93.8	Strong agreement
	Train spiritual care providers (e.g., role-play case scenarios, simulate empathic responses, generate case studies)	76	7	3	0	77.6	Moderate agreement	79	4	3	1	86.1	Strong agreement
	Adapt spiritual resources and advise on diverse traditions and cultures (e.g., adjust prayers or meditations to fit patient background, provide accurate information about diverse spiritual traditions, rituals, and values to support culturally appropriate care)	77	6	3	0	76.6	Moderate agreement	78	5	3	1	85.9	Strong agreement

	Support decision-making and prioritization in spiritual care (e.g., triage patients, flag urgent cases, generate referrals, suggest interventions, ensure timely follow-up or immediate human support)	78	5	3	0	75.6	Moderate agreement	75	8	3	1	92.0	Strong agreement
	Create legacy tasks and advance directives (e.g., write ethical wills, life reviews, or farewell letters based on patient inputs)	76	7	3	1	73.7	Moderate agreement	77	6	3	1	83.1	Strong agreement
	Identify patients which may need spiritual care (e.g., conduct screening or intake questionnaires, take a spiritual history, analyze records for patterns, anticipate needs)	78	5	3	1	69.2	Moderate agreement	79	4	3	1	87.3	Strong agreement
	Monitor spiritual well-being over time and identify patients at risk of distress (e.g., analyze patient communication for themes like fear, grief, hope, or meaning)	70	13	3	1	68.6	Moderate agreement	70	13	3	1	85.7	Strong agreement
	Conduct spiritual assessments (e.g., guide patients through in-depth questionnaires, record and organize their responses, and summarize findings in standardized formats)	74	9	3	1	62.2	Moderate agreement	74	9	3	1	81.1	Strong agreement
	Create supportive spaces (e.g., host peer-support groups, moderate online communities, create immersive virtual prayer and meditation environments)	69	14	2	1	43.5	No consensus	66	17	3	1	59.1	No consensus
	Direct patient engagement (e.g., engage patients in conversation, provide words of care and comfort, and conduct remote check-ins)	76	7	2	1	42.1	No consensus	72	11	3	1	62.5	Moderate agreement
	Conduct ritual tasks (e.g., guide meditations, recite or adapt prayers, lead ceremonies)	76	7	2	1	42.1	No consensus	75	8	2	2	45.3	No consensus
AI can <u>substitute</u> the following tasks (i.e. perform them instead of a human provider): (Likert)	Summarize and classify information (e.g., build taxonomies of spiritual needs, condense patient stories, summarize prayers or scriptures)	78	5	3	0	79.5	Moderate agreement	77	6	3	1	92.2	Strong agreement
	Administrative and routine tasks (e.g., schedule patients, recommend follow-ups, produce reports)	79	4	3	0	78.5	Moderate agreement	79	4	4	1	94.9	Strong agreement
	Conduct research in spiritual care (e.g., analyze encounter data, identify trends, produce summaries for studies)	79	4	3	1	73.4	Moderate agreement	75	8	3	1	88.0	Strong agreement
	Documentation and care planning (e.g., draft chart notes, generate patient profiles, pre-fill care plans)	78	5	3	1	69.2	Moderate agreement	74	9	3	1	87.8	Strong agreement
	Create legacy tasks and advance directives (e.g., write ethical wills, life reviews, or farewell letters based on patient inputs)	76	7	3	1	59.2	No consensus	70	13	3	2	70.0	No consensus

	Identify patients which may need spiritual care (e.g., conduct screening or intake questionnaires, take a spiritual history, analyze records for patterns, anticipate needs)	73	10	3	1	58.9	No consensus	76	7	3	1	81.6	Strong agreement
	Support decision-making and prioritization in spiritual care (e.g., triage patients, flag urgent cases, generate referrals, suggest interventions, ensure timely follow-up or immediate human support)	74	9	3	1	54.1	No consensus	72	11	3	2	73.6	No consensus
	Train spiritual care providers (e.g., role-play case scenarios, simulate empathic responses, generate case studies)	76	7	3	1	53.9	No consensus	75	8	3	1	70.7	Moderate agreement
	Monitor spiritual well-being over time and identify patients at risk of distress (e.g., analyze patient communication for themes like fear, grief, hope, or meaning)	73	10	2	1	49.3	No consensus	68	15	3	1	72.1	Moderate agreement
	Adapt spiritual resources and advise on diverse traditions and cultures (e.g., adjust prayers or meditations to fit patient background, provide accurate information about diverse spiritual traditions, rituals, and values to support culturally appropriate care)	72	11	2	1	45.8	No consensus	72	11	3	1	73.6	Moderate agreement
	Conduct spiritual assessments (e.g., guide patients through in-depth questionnaires, record and organize their responses, and summarize findings in standardized formats)	77	6	2	1	44.2	No consensus	74	9	3	1	59.5	No consensus
	Direct patient engagement (e.g., engage patients in conversation, provide words of care and comfort, and conduct remote check-ins)	77	6	2	2	26.0	No consensus	71	12	2	2	39.4	No consensus
	Conduct ritual tasks (e.g., guide meditations, recite or adapt prayers, lead ceremonies)	79	4	2	2	25.3	No consensus	76	7	2	1	32.9	Moderate disagreement
	Create supportive spaces (e.g., host peer-support groups, moderate online communities, create immersive virtual prayer and meditation environments)	74	9	2	1	18.9	Strong disagreement	65	18	2	1	38.5	Moderate disagreement

Table S6. Perception of current capabilities and future potential of AI for specific spiritual care-related tasks, in order of % agree.

	Item	Weighted score	Count	Mean rank (explicit)	SD	Consensus
The potential benefits of AI use by spiritual care providers are: (Ranking)	Less administrative work	139	40	2.52	1.60	No consensus
	Improved screening and triage	108	31	2.52	1.48	High priority
	Improved documentation	107	38	3.18	1.35	High priority
	Better access to information and resources	99	35	3.17	1.25	High priority
	Improved research	93	32	3.09	1.28	High priority
	Better summaries of patient data	91	26	2.50	1.27	High priority
	Greater workplace efficiency	80	26	2.92	1.65	No consensus
	Improved care taxonomies	58	16	2.38	1.09	Moderate priority
	Improved spiritual assessments	44	13	2.62	1.39	Moderate priority
	More patient referrals	40	11	2.36	1.21	Moderate priority
	Improved quality of patient data	37	15	3.53	1.36	Moderate priority
	Expanded access and availability	36	14	3.43	1.60	No consensus
	Improved care planning	35	14	3.50	1.09	Moderate priority
	Better-informed clinical decisions	34	11	2.91	1.30	Moderate priority
	Better tools for self-guided care	33	16	3.94	1.12	Moderate priority
	More personalized care	28	8	2.50	1.41	Low priority
	Deeper patient understanding	28	9	2.89	1.05	Low priority
	Increased innovation	25	10	3.50	1.08	Low priority
	Lower cost of care	23	8	3.12	1.89	No consensus
	Reduced stigma in care-seeking	20	7	3.14	1.35	Low priority
	Closer interprofessional collaboration	20	10	4.00	0.94	Low priority
	More creative caregiving	15	6	3.50	1.97	No consensus
	Fewer provider errors and biases	14	4	2.50	1.73	No consensus

	Better patient communication	13	5	3.40	1.52	No consensus
	Safer online communities	12	4	3.00	1.63	No consensus
	Improved perspective taking	9	3	3.00	1.73	No consensus
	Improved prayer practices	3	2	4.50	0.71	Lowest priority
	There are no potential benefits of AI use by spiritual care providers	1	1	5.00	0.00	Lowest priority
	Improved ritual practices	0	0	0.00	0.00	Lowest priority
The potential risks of AI use by spiritual care providers are: (Ranking)	Loss of human connection	122	39	2.87	1.45	High priority
	Dehumanization of spiritual care	117	34	2.56	1.48	High priority
	Uncritical use of AI	103	28	2.32	1.25	High priority
	Bias and discrimination	79	27	3.07	1.33	High priority
	Artificial and inauthentic care	77	23	2.65	1.23	High priority
	Errors and hallucinations	63	23	3.26	1.39	High priority
	Oversimplified and trivialized patient care	60	17	2.47	1.28	High priority
	Lack of human oversight	51	15	2.60	1.30	Moderate priority
	Harm and abuse	49	13	2.23	1.42	Moderate priority
	Generic and impersonal care	40	14	3.14	1.70	No consensus
	Misaligned care priorities (e.g., AI systems are optimized for cost, not patient needs)	39	16	3.56	1.41	Moderate priority
	Devaluation of human providers	36	13	3.23	1.09	Moderate priority
	Replacement of chaplains	34	13	3.38	1.50	No consensus
	Environmental impact	30	11	3.27	1.42	Moderate priority
	Use of AI to cut costs	29	9	2.78	1.79	No consensus
	Erosion of professional expertise	29	11	3.36	1.50	No consensus
	Lack of privacy and confidentiality	28	11	3.45	1.57	No consensus
	Invalid or hollow rituals	27	9	3.00	1.32	Low priority
	System security vulnerabilities	26	9	3.11	1.17	Low priority

Erosion of patient trust	26	9	3.11	1.17	Low priority
Mis- and disinformation	24	8	3.00	1.20	Low priority
Inappropriate use in sacred contexts	22	8	3.25	1.58	No consensus
Accountability concerns (e.g., who is responsible when harm occurs)	21	8	3.38	1.60	No consensus
Lack of disclosure and consent to AI use	19	6	2.83	1.47	Low priority
Inequities and access barriers	16	7	3.71	1.60	No consensus
Spiritual projection (e.g., patients treating AI as a divine being)	15	8	4.12	0.83	Low priority
Lack of transparency (e.g., regarding AI-involvement in patient communication)	13	4	2.75	1.50	No consensus
Poor crisis management	12	5	3.60	0.89	Lowest priority
Plagiarism and academic dishonesty	12	3	2.00	1.73	No consensus
Human care providers are left behind	10	4	3.50	1.29	Lowest priority
Undermining of training and education	9	6	4.50	0.84	Lowest priority
Other risks	5	2	3.50	2.12	No consensus
Legal liabilities	2	2	5.00	0.00	Lowest priority
There are no potential risks of AI use by spiritual care providers	0	0	0.00	0.00	Lowest priority

Table S7. Perceived risks and benefits of AI use in spiritual care, in order of weighted score.

		**Count**	**%**
In your view, do the potential benefits of AI in spiritual care outweigh the potential risks? (Single choice)	The benefits outweigh the risks	38	45.8
	The benefits and risks are about equal	18	21.7
	Don't know/it depends	15	18.1
	The risks outweigh the benefits	12	14.5

Table S8. Perceived risk-benefit tradeoff of AI use in spiritual care, in order of count.

		n	Don't Know	Mdn	IQR	Agree (%)	Consensus
Do you believe effective spiritual care requires the care provider to have human consciousness? (Likert)	Human presence gives spiritual care a moral, spiritual or emotional depth that AI cannot replicate	78	5	4	1	94.9	Strong agreement
	The empathy and presence needed for spiritual care can only arise from genuine human connection	75	8	3	1	92.0	Strong agreement
	Human and AI contributions to spiritual care should be judged by their complementarity, not competition	78	5	3	1	91.0	Strong agreement
	Spiritual care should be judged by its benefit to the patient, not by whether the caregiver is human	73	10	3	1	60.3	Moderate agreement
Should AI-provided spiritual care always involve or connect back to a human caregiver? (Likert)	AI should only be used as a support tool, with final responsibility resting with a human	79	4	4	1	94.9	Strong agreement
	AI should never be used without human involvement in sensitive or high-risk situations	79	4	4	0	93.7	Strong agreement
	AI-provided spiritual care should always be supervised by a human caregiver	79	4	4	1	92.4	Strong agreement
	A hybrid approach, combining AI support with human oversight, is the most appropriate model	77	6	3	1	83.1	Strong agreement
	AI may provide care independently for simple or routine tasks	77	6	3	1	71.4	Moderate agreement
	Patients should be able to choose whether their AI encounter connects back to a human caregiver	77	6	3	1	57.1	No consensus
	The need for human involvement depends on the patient's preference	78	5	2	1	42.3	No consensus
The following theological or ethical objections to the use of AI in spiritual care should be considered: (Likert)	Substituting AI for embodied human presence undermines the sacred dimension of care	73	10	3	1	86.3	Strong agreement
	AI lacks the soul, consciousness, or spiritual authority needed for authentic care	73	10	3	1	82.2	Strong agreement
	Reliance on AI weakens the communal dimension of faith and belonging	76	7	3	1	75.0	Moderate agreement
	AI contributes to environmental harm and undermines stewardship of creation	70	13	3	2	71.4	No consensus
	Religious rituals and sacraments performed by AI are invalid or inappropriate	65	18	3	2	70.8	No consensus
	Using AI to simulate empathy is ethically problematic, even if it comforts patients	74	9	3	2	68.9	No consensus
	AI use in spiritual care raises theological concerns about creation, Imago Dei, or human uniqueness	76	7	3	1	68.4	Moderate agreement
	AI use reflects an instrumental, efficiency-driven rationality, not patient needs	72	11	3	1	56.9	No consensus
	Promotion of AI in spiritual care is based on hype or overpromising, not on patient needs	69	14	2	1	44.9	No consensus

	AI use in spiritual care dehumanizes patients and/or caregivers	74	9	2	1	44.6	No consensus
	Decisions about AI in spiritual care should be made by religious authorities	71	12	2	2	40.8	No consensus
	AI use in spiritual care brings serious harm, including spiritual trauma	66	17	2	1	37.9	Moderate disagreement
	There are no theological or ethical objections to AI use in spiritual care if it supports patients effectively	78	5	2	2	25.6	No consensus
The following limits should be placed on the use of AI in spiritual care: (Likert)	Implementation of AI in spiritual care must be based on evidence of safety and effectiveness	82	1	4	1	100	Strong agreement
	The introduction of AI in spiritual care must follow principles of responsible and cautious innovation	82	1	4	1	100	Strong agreement
	Extra safeguards are needed when AI is used with vulnerable patients	83	0	4	1	100	Strong agreement
	Care planning contributions generated by AI require human approval before use	83	0	3	1	100	Strong agreement
	Strict standards of data protection and privacy are required for all uses of AI in spiritual care	82	1	4	0	98.8	Strong agreement
	Decisions about the use of AI in spiritual care should involve consultation with patients, providers, and faith communities	81	2	3	1	98.8	Strong agreement
	Clear governance and oversight structures are required for the use of AI in spiritual care	82	1	4	1	97.6	Strong agreement
	AI can be used in spiritual care education only under clear ethical guidelines	79	4	3	1	97.5	Strong agreement
	The use of AI in spiritual care requires personal reflection by providers	78	5	3	1	97.4	Strong agreement
	Ethical and moral decision-making in spiritual care must remain under human responsibility	80	3	4	1	96.3	Strong agreement
	AI in spiritual care must only assist human caregivers, not replace them	79	4	4	1	96.2	Strong agreement
	Assessments supported by AI in spiritual care must always be reviewed by a human caregiver	81	2	4	1	95.1	Strong agreement
	Patients and providers must be clearly informed whenever AI is used in spiritual care	77	6	4	1	94.8	Strong agreement
	AI in spiritual care should only be applied in ways that promote equity and social justice	80	3	3	1	92.5	Strong agreement
	AI involvement in spiritual care requires prior patient consent	76	7	4	1	90.8	Strong agreement
	Simulation of God or divine beings by AI is never acceptable in spiritual care	72	11	4	1	90.3	Strong agreement
	AI must not perform sacraments or lead rituals	68	15	3	1	86.8	Strong agreement

	AI-enabled chatbots must be customized to reflect the patient's faith or spiritual tradition	68	15	3	1	75.0	Moderate agreement
	AI should only be used for administrative tasks; direct, patient-facing spiritual care tasks should remain the responsibility of humans	75	8	3	2	64.0	No consensus
	AI must never be used during crises	61	22	3	1	50.8	No consensus
	No use of AI to generate or perform prayer	68	15	2	1	44.1	No consensus
	AI should not be used in spiritual care at all	80	3	2	1	10.0	Strong disagreement
	No limits or requirements are needed or possible	80	3	1	0	6.3	Strong disagreement

Table S9. Theological and ethical considerations of AI use in spiritual care, in order of % agree.

		**Weighted score**	**Count**	**Mean rank (explicit)**	**SD**	**Consensus**
Spiritual care providers would need the following professional competencies to safely and effectively use AI in patient care: (Ranking)	Judge when AI is applicable and identify boundaries of its use	153	49	2.88	1.33	High priority
	Safeguard patient privacy and data in AI contexts	124	37	2.65	1.48	High priority
	Assess and mitigate risks associated with AI use	119	35	2.60	1.35	High priority
	Exercise theological and ethical discernment in relation to AI use	117	36	2.75	1.30	High priority
	Engage in self-reflection on how AI use affects care practice	100	35	3.14	1.63	No consensus
	Ability to operate AI tools and understand their functions	99	32	2.91	1.51	No consensus
	Evaluate AI performance and identify limitations	79	28	3.18	1.25	Moderate priority
	Apply core spiritual care competencies in contexts that include AI	77	25	2.92	1.47	Moderate priority
	Appropriately disclose AI use to patients	74	26	3.15	1.52	No consensus
	Maintain chaplain authority and professional identity when using AI	69	26	3.35	1.23	Low priority
	Detect and appropriately respond to bias in AI outputs	63	24	3.38	1.31	Low priority
	Conduct and use research to inform safe and effective AI use	51	19	3.32	1.53	No consensus
	Exercise leadership in guiding responsible AI integration	44	18	3.56	1.50	No consensus
	Design and adapt prompts for AI systems	40	13	2.92	1.50	No consensus
	Ensure equitable access to AI tools and care resources	34	11	2.91	1.30	Lowest priority
	Other competencies	2	1	4.00	0.00	Lowest priority

Table S10. Required professional competencies to safely and effectively use AI in spiritual care, in order of weighted score.
